# Supplementary material for: An Integrated Genomic Strategy to Identify CHRNB4 as a Diagnostic/Prognostic Biomarker for Targeted Therapy in Head and Neck Cancer
Source: Cancers (Basel). 2020 May 22;12(5):1324. doi: 10.3390/cancers12051324 (PMC7281299; doi:10.3390/cancers12051324)
Supplement: Supplementary file 1 [file cancers-12-01324-s001.zip › cancers-811173-suppl proof.docx]

Supplementary Materials: An Integrated Genomic Strategy to Identify CHRNB4 as a Diagnostic/Prognostic Biomarker for Targeted Therapy in Head and Neck Cancer


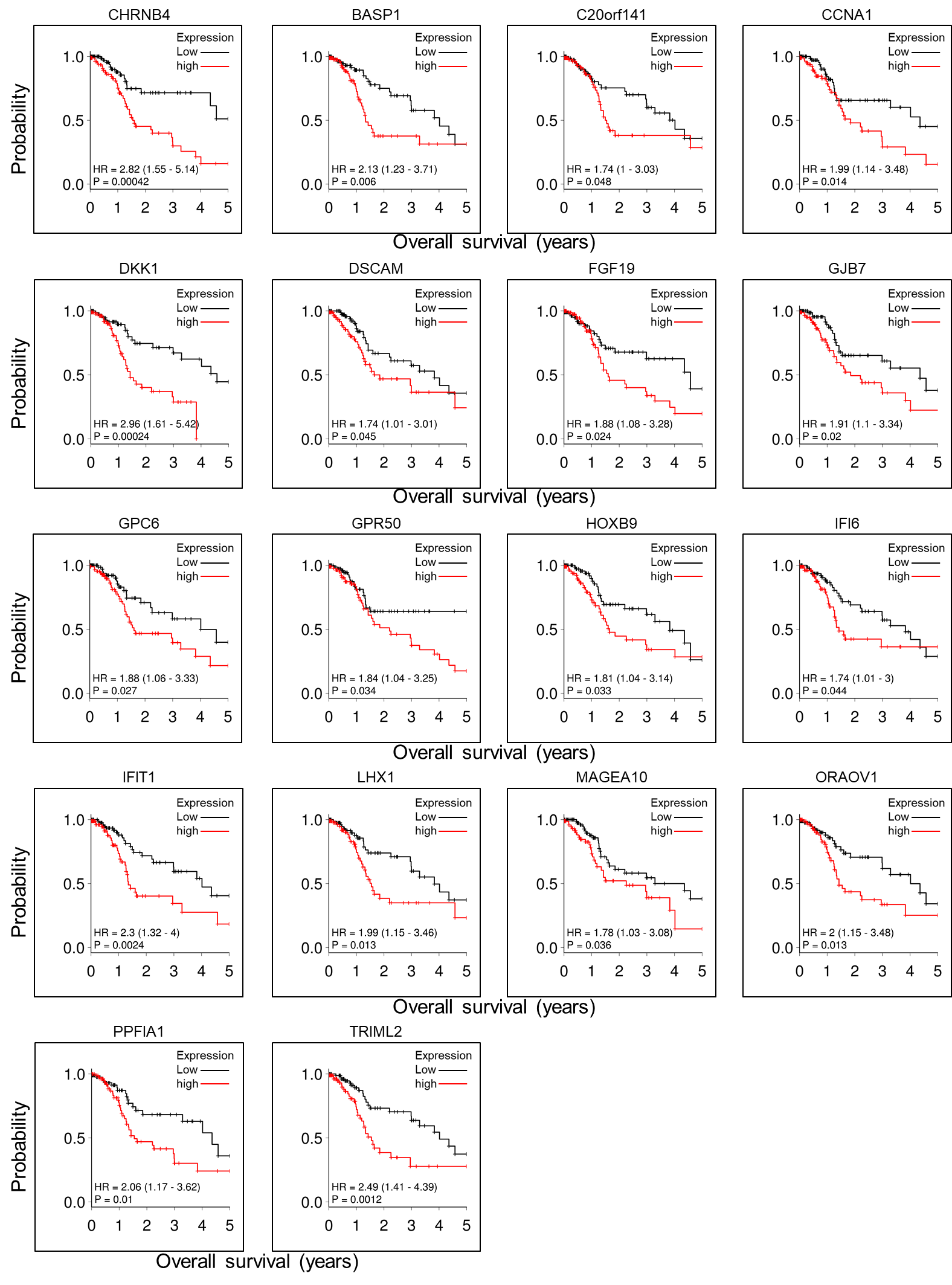


**Figure S1.** Kaplan-Meier plots of overall survival (OS) of 18 adverse genes. Smoking HNSCC patients were stratified into two subgroups by gene expression median value of each gene. Patients with gene expression value lower than median value were displayed in black line, otherwise red line. Statistical significances of OS between two subgroups was described by the log-rank test *p* < 0.05. Hazard ratio (HR) was computed by Cox proportional hazards regression, and the 95% confidence intervals for each gene are showed in parentheses.


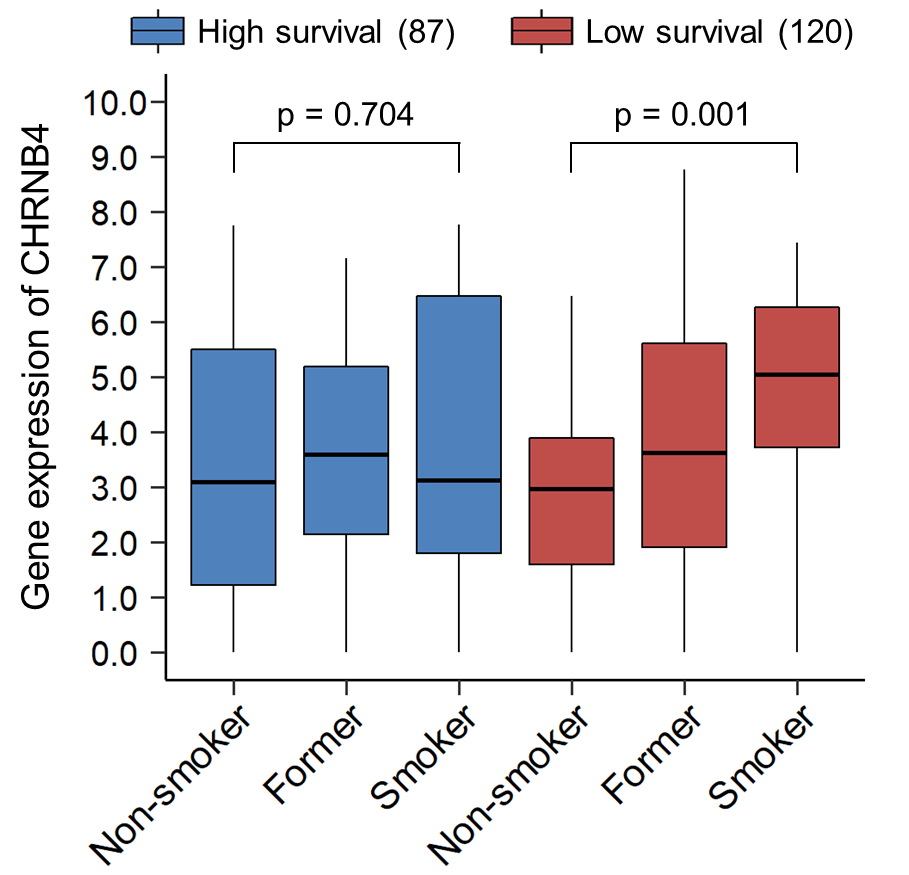


**Figure S2**. Boxplot of CHRNB4 gene expression of high- and low-survival subgroups with different smoking history. Patients were classified into ‘low survival group’ (adverse prognosis, red box) and ‘high survival group’ (favourable prognosis, blue box). Patients who had died within the time less than 75% of total follow-up duration, were stratified into low survival group (red box). Otherwise, patients who had censored alive for more than 75% of total follow-up time, were stratified into high survival group (blue box). The two-sided p values were calculated by Student’s t-test.

**Table S1.** Characteristics of the study patients from TCGA.

| **Patients, No.** | | | | |
| --- | --- | --- | --- | --- |
| Characteristic | Total | Smoker | Non-smoker | Former |
| Smoking status |  |  |  |  |
| Smoker | 175 | NA | NA | NA |
| Non-smoker | 117 | NA | NA | NA |
| Former | 212 | NA | NA | NA |
| Mortality |  |  |  |  |
| Alive | 345 | 120 | 88 | 137 |
| Dead | 159 | 55 | 29 | 75 |
| Age (year) |  |  |  |  |
| <50 | 79 | 29 | 32 | 18 |
| 50-65 | 255 | 105 | 49 | 101 |
| >65 | 170 | 41 | 36 | 93 |
| Sex |  |  |  |  |
| Male | 373 | 140 | 67 | 166 |
| Female | 131 | 35 | 50 | 46 |
| Drinker |  |  |  |  |
| Yes | 345 | 138 | 60 | 147 |
| No | 149 | 32 | 55 | 62 |
| Not Available | 10 | NA | NA | NA |
| Cancer stage (TNM) |  |  |  |  |
| Stage I & Stage II | 112 | 29 | 35 | 48 |
| Stage III | 103 | 34 | 24 | 45 |
| Stage IVA,B,C | 275 | 104 | 56 | 115 |
| Not Available | 14 | 8 | 2 | 4 |
| HPV status p16 |  |  |  |  |
| Positive | 37 | 11 | 11 | 15 |
| Negative | 72 | 31 | 11 | 30 |
| Not Available | 395 | NA | NA | NA |

Abbreviation: NA, not applicable.

We analyzed the multivariate and univariate Cox proportional hazards regression to examined the prognostic predicting power of CHRNB4-signature and clinicopathological factors (e.g., gender, age, alcohol consumption, primary tumour stage, regional lymph nodes metastasis and clinical TNM stage) for smoking patients (Table S2). The results of univariate analysis showed that CHRNB4 signature (CHRNB4-high smokers vs. CHRNB4-low smokers) and regional lymph node (N+ vs. N0) were significantly associated with 5-year overall survival for smoking HNSCCs. And the results of multivariate analysis displayed that CHRNB4 signature and regional lymph node were significantly independent prognostic factors for smoking HNSCCs (CHRNB4 signature: HR [95% CI], 2.94 [1.59-5.43]; Wald test, *p* = 0.00056; lymph node: HR [95% CI], 2.49 [1.16-5.34]; Wald test, *p* = 0.019).

**Table S2.** Univariate and multivariate Cox proportional hazards regression analysis of 5-year overall survival in smoking HNSCC patients (*n* = 175).

| **Variables (HNSCC Smokers, *n* = 175)** | **Univariate** | | **Multivariate** | |
| --- | --- | --- | --- | --- |
|  | **HR (95% CI)** | **Wald Test, *p*** | **HR (95% CI)** | **Wald Test, *p*** |
| CHRNB4 signature (CHRNB4-high) | 2.82 (1.55-5.14) | 0.00072 | 2.94 (1.59-5.43) | 0.00056 |
| Gender (male) | 0.65 (0.35-1.21) | 0.17 | 0.88 (0.45-1.71) | 0.7 |
| Age (>60) | 0.77 (0.43-1.38) | 0.38 | 0.94 (0.51-1.73) | 0.85 |
| Alcohol (Yes) | 1.26 (0.61-2.59) | 0.53 | 0.82 (0.38-1.77) | 0.61 |
| Primary tumour (T3 & T4) | 0.98 (0.5-1.92) | 0.96 | 1.21 (0.35-4.14) | 0.76 |
| Regional lymph node (N+) | 1.95 (1.09-3.48) | 0.025 | 2.49 (1.16-5.34) | 0.019 |
| Clinical stage (stage III & IV) | 1.06 (0.5-2.27) | 0.87 | 0.50 (0.11-2.31) | 0.37 |

| 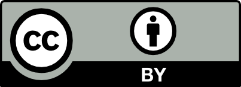 | © 2020 by the authors. Submitted for possible open access publication under the terms and conditions of the Creative Commons Attribution (CC BY) license (http://creativecommons.org/licenses/by/4.0/). |
| --- | --- |
