# Supplementary figures and images for: An Integrated Genomic Strategy to Identify CHRNB4 as a Diagnostic/Prognostic Biomarker for Targeted Therapy in Head and Neck Cancer
Source: Cancers (Basel). 2020 May 22;12(5):1324. doi: 10.3390/cancers12051324 (PMC7281299; doi:10.3390/cancers12051324)

Figure 6A


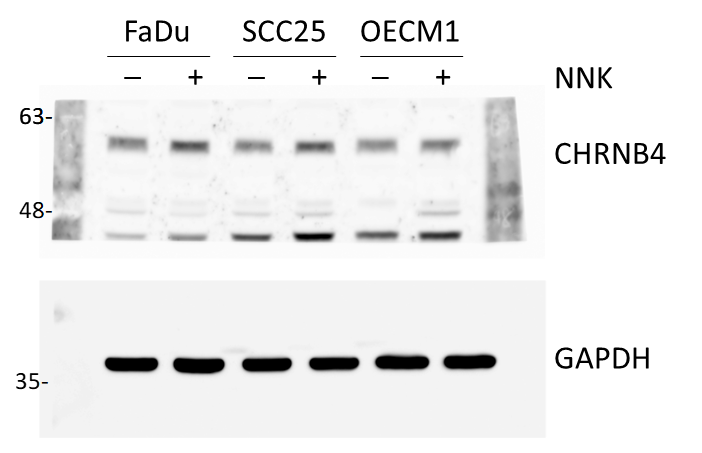


Figure 6B


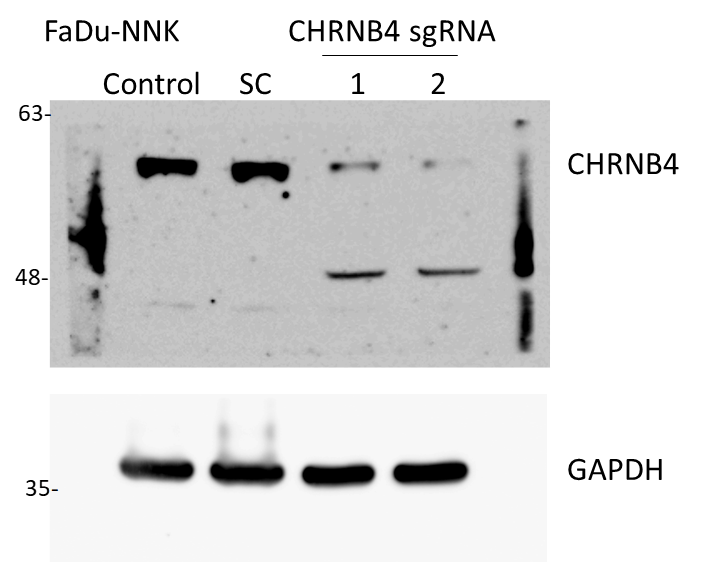


Figure 6C


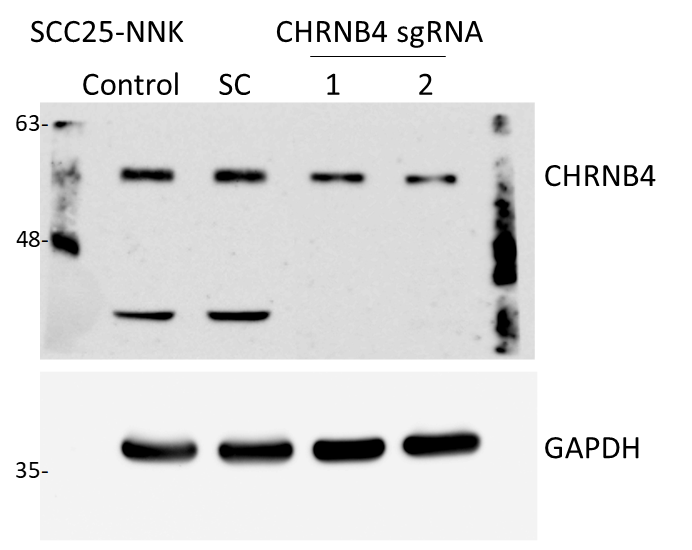

Supplement: Supplementary file 1 [file cancers-12-01324-s001.zip › Supplementary Data 7. Western blot original data.docx]
